# Gingival Overgrowth Leading to the Diagnosis of Familial Tuberous Sclerosis Complex

**DOI:** 10.1155/2016/8195321

**Published:** 2016-01-13

**Authors:** Parth Purwar, Sagar Sareen, Vaibhav Sheel, Abhishek Gupta, Uzma Ansari, Patel Umeshbhai Becharbhai, Manisha Dixit, Amitesh Bhargava, Rajiv Ratan Singh Yadav, Utkarsh Bansal, Jaya Dixit

**Affiliations:** ^1^Department of Periodontology, Faculty of Dental Sciences, King George's Medical University, Lucknow, Uttar Pradesh, India; ^2^Ankur Diagnostic Center, Lakhimpur Road, Gola Gokaran Nath, Kheri, Uttar Pradesh 262802, India; ^3^Department of Community Medicine, Hind Institute of Medical Sciences, Barabanki, Uttar Pradesh, India; ^4^Emergency Medicine, Dr. Ram Manohar Lohia Institute of Medical Sciences, Uttar Pradesh, India; ^5^Department of Paediatrics, Hind Institute of Medical Sciences, Barabanki, Uttar Pradesh, India

## Abstract

Tuberous sclerosis complex (TSC), a neurocutaneous syndrome, is characterized by the development of benign tumours affecting different body systems. We herein present a report of a 40-year-old female patient presenting with dental enamel pits and localized gingival overgrowth that eventually lead to the diagnosis of a case of familial TSC. Diagnosis of familial TSC by comprehensive oral examination and detection of oral manifestations proved to be inevitable as it resulted in institution of appropriate treatment strategies and genetic counselling of the affected family.

## 1. Introduction

Tuberous sclerosis complex (TSC) shows variable clinical expressivity with oral manifestations observed in less than 10% of the cases and familial inheritance pattern perceived in only 30% of the cases reported [1, 2]. The most commonly noted oral manifestations in TSC are presence of oral fibromas, gingival hyperplasia, and enamel hypoplasia in the form of dental enamel pitting. This case highlights the importance of oral manifestations in the diagnosis of a case of familial TSC.

## 2. Case Presentation

**Figure 1:**
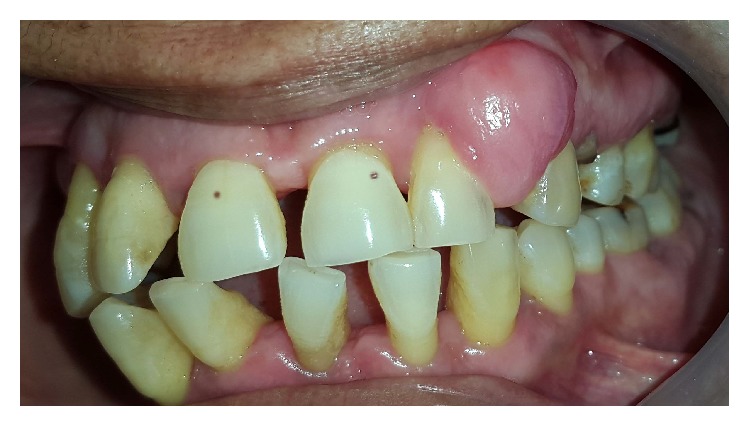
Preoperative facial view showing gingival overgrowth in upper left premolar region.

A 40-year-old female patient reported to the Department of Periodontology of King George's Medical University, Lucknow, in April 2015, with a chief complaint of painless gingival overgrowth in left upper posterior teeth region (Figure 1). On further elaboration of her complaint she revealed that initially the overgrowth was localized and freely movable. However, in last 6 months, the growth has progressively increased in size with involvement of multiple teeth leading to compromised aesthetics and poor masticatory efficiency.

On thorough physical examination the propositor did not show any signs or symptoms of cardiovascular, endocrine, respiratory, immune, or musculoskeletal disorders. On further elaboration of the medical history, the propositor's elder brother revealed occurrence of sporadic epileptic episodes since childhood with eventual reoccurrences occasionally which was treated with irregular use of antiseizure drugs. Upon extra oral examination some nodular lesions were also seen around the nasal and perinasal area. On intraoral examination a localized diffuse gingival overgrowth involving marginal, papillary, and attached labial gingiva was seen extending from the left maxillary premolar to left maxillary canine. The overlying gingiva was pink, nonerythematous, and of semifirm consistency with loss of stippling. Some form of enamel hypoplasia/pitting on vestibular surface of upper anterior teeth was also observed which further jeopardized her esthetics. The propositor's attendant also gave history of delayed eruption of the permanent teeth. The propositor also disclosed that she had been a tobacco chewer since her teenage years. Based on the clinical presentation and history of intake of antiseizure drugs a provisional diagnosis of drug induced gingival overgrowth was made and a tailored treatment plan was constituted which included institution of oral hygiene instructions and initiation of phase I periodontal therapy. During second recall visit, on reviewing the patient history, the sporadic use of antisiezure drugs made us think on the lines of associated syndromic conditions.

**Figure 2:**
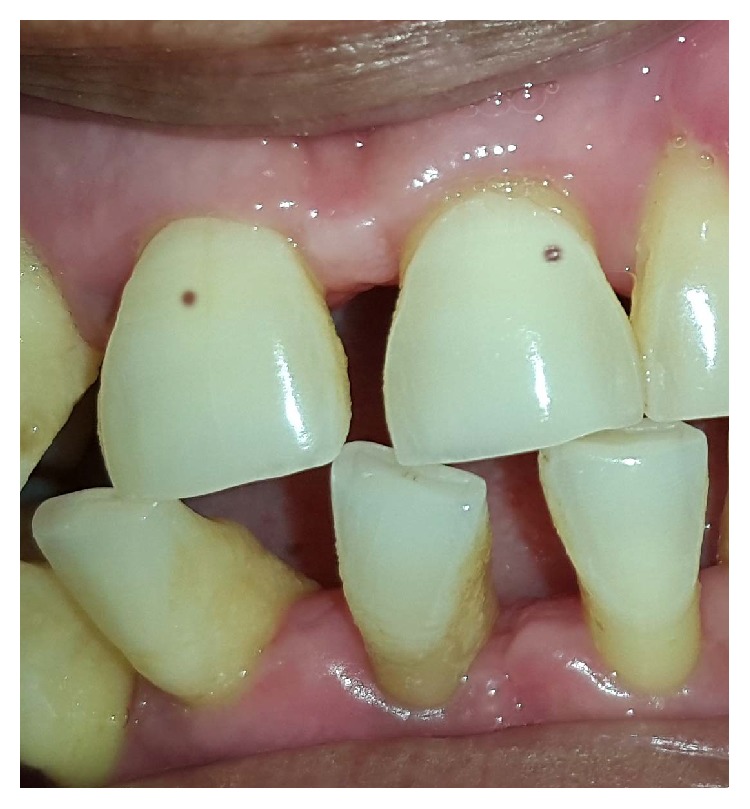
Enamel pitting in upper permanent central incisor region.

**Figure 3:**
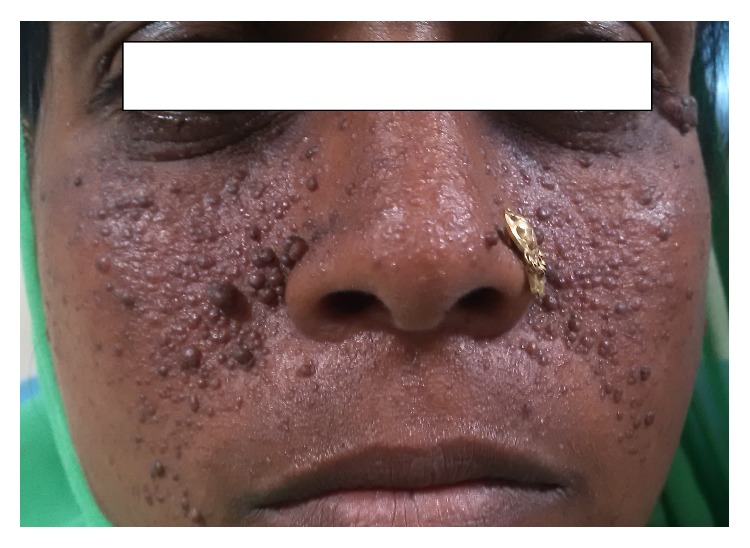
Multiple angiofibromas affecting the nasolabial folds and nose.

On reexamining the patient, the comorbid appearance of small enamel pits (Figure 2) and gingival overgrowth indicated the possible clinical working hypothesis of tuberous sclerosis complex (TSC). Comprehensive dermatological examination was performed to look for the cutaneous manifestations, if any, to potentiate the clinical diagnosis of TSC. Examination by dermatologist revealed presence of nodular lesions in nasal and perinasal area which appeared in a characteristic butterfly fashion (Figure 3). Further questioning also revealed that other family members of the propositor had similar lesions on face and were later on invited to undergo oral and dermatological examination to confirm the familial nature of the disease. Six members of the family who were in direct blood relation including her father and brother showed ungual lesions, hypopigmented macules in perinasal area, and dental enamel pits on upper front teeth. The familial findings indicated an autosomal dominant inheritance pattern of the disease in the present case. To further affirm the diagnosis, the propositor was submitted to the imaging analysis. Ultrasonography of abdomen showed viscera to be normal; however both the kidneys were poorly visualized. Furthermore, computed tomography (CT) of whole abdomen showed that both the kidneys were totally replaced by heterogeneous mass lesion showing fat components and thick septations. The affection was found to be more prominent in the left kidney than in the right one. The renal lesion in left side measured approx 18 × 15 cm while right renal mass measured 9 × 7 cm. Only small renal parenchyma was appreciated at the lower pole of both the kidneys. A written informed consent for the photographs being used was taken from the patient.

Based on the clinical and radiological findings along with the familial inheritance pattern the diagnosis of autosomal dominant tuberous sclerosis complex (TSC) was established consisting of a triad of bilateral renal angiomyolipoma, pancreatic angiomyolipoma, and skin angiofibroma along with intraoral findings as gingival overgrowth and enamel pits.

## 3. Investigations

**Figure 4:**
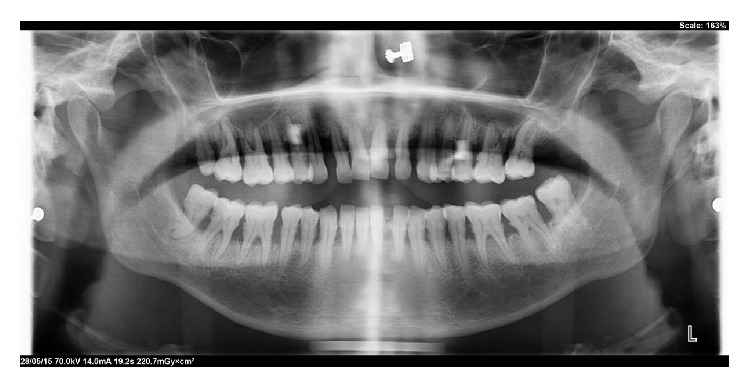
Orthopantomogram showing normal bone morphology.

Routine haematological investigations were conducted and were found to be within the reference range. Imaging analysis included USG and CT scan of whole abdomen. Kidney function test was normal and asymptomatic. The urine analysis did not reveal any significant finding. Echocardiography (ECG) and fundoscopy examinations were found to be nonsignificant. Magnetic resonance imaging (MRI) could not be performed due to financial constraints. Oral imaging revealed normal bone morphology as evident by orthopantamograms (Figure 4).

## 4. Differential Diagnosis

Other forms of neurocutaneous syndromes include type 1 and type 2 neurofibromatosis, Sturge-Weber syndrome, ataxia-telangiectasia and von Hippel Landau disease, and tuberous sclerosis complex [3, 4]. Facial and oral papules/nodules can also be seen in Cowden syndrome, Birt-Hogg-Dube syndrome, and multiple endocrine neoplasia type-1 [3, 4]. Enamel pits can be associated with multiple conditions such as pitted hypoplastic amelogenesis imperfecta, vitamin-D dependent rickets, pseudohypoparathyroidism, and junctional epidermolysis bullosa [4].

## 5. Treatment

During initial treatment visit, gentle scaling and root planing were done along with institution of oral hygiene instructions. Plaque control measures lead to the improvement in the periodontal status but the lesion did not resolve completely on subsequent visits. For the same reason, complete excision till periosteum was planned with surgical scalpel under local anaesthesia. An informed written consent was taken from the propositor. Under local anesthesia full thickness mucoperiosteal flap was reflected involving permanent canine and premolars. Root planing and flap curettage were done and irrigated with saline. Flap was repositioned with figure of eight sutures and noneugenol surgical periodontal dressing was placed. Postoperative instructions as well as indicated medications were prescribed. The excised gingival tissue appeared pale pink and fibrous and did not differ from any usual enlarged gingival tissue. Also, the patient has been referred to the department of urology for laproscopic examination for diagnosing renal lesions. For hamartomatous lesions on face, cosmetic treatments have been advised ranging from cryosurgery to lasers.

## 6. Outcome and Follow-Up

The patient was recalled after 10 days for follow-up. The periodontal dressings were removed and healing took place uneventfully. Oral hygiene instructions were reinforced. No recurrence has been observed till now. The genetic counselling for the propositor and affected family members has been rendered from the Centre for Advanced Research, KGMU.

## 7. Discussion

There is a paucity of literature in which the diagnosis of tuberous sclerosis complex (TSC) was based on the oral findings; the aim of this case report is to emphasize oral manifestations for the diagnosis of familial case of TSC.

TSC or Bourneville's disease (MIM 191100) is an autosomal dominant inherited disorder affecting multiple organs such as skin, adnexa, CNS, heart, and kidneys [2–5]. TSC was first described by Von Recklinghausen and later by Pringle [6].

In this case report the propositor presented with findings of multiple angiofibromatous hamartomatous lesions distributed in a butterfly wing-like pattern around malar region, infrequent occurrence of seizures and renal and pancreatic angiomyolipomas along with enamel pitting, and localized gingival overgrowth. TSC has been associated with epilepsy in 90% of cases, with Koenen tumours in 15–20% and angiofibromas in 70% of cases, and with renal angiomyolipomas in 50% of the cases [7, 8]. The extraoral and intraoral findings herein presented are in agreement with the clinical diagnostic criteria of TSC (major and minor features) as described by Northrup and Krueger in 2013 [9]. However the genetic diagnostic criteria could not be confirmed due to financial constraints. The incidence of oral fibromas in TSC cases varies between 50 and 69% with an average diameter of 5 mm [10]. The aggressivity of such oral lesions depends upon the presence of local irritating factors. Herein, the gingival overgrowth was carefully excised with the help of scalpel after performing phase 1 periodontal therapy. No reoccurrence has been noted till 6 months follow-up owing to the effectiveness of the treatment. Although gingival overgrowth can also be caused by the use of antiseizure drugs as in the present case, the pattern of gingival overgrowth induced by antiseizure drug was not found in the present case was not found in the present case.

Angiofibromas in TSC have been frequently reported in upper anterior region and rarely on lip, tongue, and palate. Enamel pits seen in association with TSC may be associated with high risk of dental caries. Less frequent dental findings reported in association with TSC include high arched palate, bifid uvula, hare lip and/or cleft palate, delayed eruption of teeth, and presence of diastema [11]. The mutational analysis have also become an additional diagnostic tool for the diagnosis of cases of familial TSC. Two disease determining genes have been identified, namely, TSC-1 and TSC-2. TSC-1 protein product is hamartin and TSC-2 protein product is tuberin. Both of these proteins are involved in cell growth and differentiation [12]. Till date no studies have found an association between TSC and oral cancer; however TSC-2 overexpression may exert antitumor effect due to its oncosuppressor genes [13, 14]. In the present case the patient had a history of epilepsy and showed cutaneous fibromas but the identification of dental enamel pits and gingival overgrowth was fundamental in the diagnosis of TSC. The patient acceptance to the treatment was also assessed by a 3-point rating scale during the follow-up visit as described by Purwar et al. [15] and the patient rated it with a score of 3 (highly satisfied) on the grounds of presurgical, surgical, and postsurgical protocol and cost-effectiveness of the treatment.

This case report illustrates the importance of identification of intraoral findings in achieving early diagnosis of TSC which leads to appropriate screening examination, treatment, and genetic counselling.

## Learning Points

- Early and accurate diagnosis of oral lesions may be helpful in diagnosis of TSC.
- TSC patients must adapt measures for careful oral and dental hygiene with regular dental visits in order to eliminate potential irritative factors.
- Oral health care professional must request a detailed medical report on the condition of the patient.
- Due to frequent pulmonary, cardiac, and renal involvement pre- and postmedication should be administered with caution.
